# Dorsal Column Bedside Examination Test: Tips for the Neurosurgical Resident

**DOI:** 10.21315/mjms2023.30.2.16

**Published:** 2023-04-18

**Authors:** Yee Hwa Khoo, Jafri Malin Abdullah, Zamzuri Idris, Abdul Rahman Izaini Ghani, Sanihah Abdul Halim

**Affiliations:** 1 Department of Neurosciences, School of Medical Sciences, Universiti Sains Malaysia, Kelantan, Malaysia; 2 Department of Neurosciences, Hospital Universiti Sains Malaysia, Kelantan, Malaysia; 3 Brain and Behaviour Cluster, School of Medical Sciences, Universiti Sains Malaysia, Kelantan, Malaysia; 4 Unit of Neurology, Department of Medicine, School of Medical Sciences, Universiti Sains Malaysia, Kelantan, Malaysia; 5 Department of Neurosurgery, Queen Elizabeth Hospital, Sabah, Malaysia

**Keywords:** proprioception, vibration, touch, sensation, neuroanatomy

## Abstract

The dorsal column medial lemniscus (DCML) system is a sensory pathway of the central nervous system; it carries sensations of soft touch, vibration, proprioception, two-point discrimination, and pressure from the skin and joints. The clinical signs of the DCML pathway lesions include loss of soft touch, vibratory sense, proprioception, discrimination sense, and a positive Rhomberg test. Diseases that affect this pathway are usually degenerative, for example, spinal cord degeneration due to vitamin B12 deficiency; it can also be affected by trauma or infarction of the posterior spinal artery causing posterior cord syndrome. This video manuscript provides a step-by-step examination technique of the dorsal column examination, specially catered for Malaysian medical students and trainees. A series of videos show the techniques for soft touch sensation examination, examination of the vibratory sense, examination of the joint position sense, examination of two-point discrimination and the Rhomberg test. We hope that students can adhere to these techniques and apply them in their daily neurological assessments.

## Introduction

The posterior column, also known as the dorsal column or dorsal column medial lemniscus (DCML) pathway, deals with two general modalities, kinaesthesia (sense of position and movement) and discriminative touch (pressure, fine touch, vibration and two-point discrimination), from our skin and joints except the head to the postcentral gyrus in the cerebral cortex and it is related to conscious proprioception (1).

The principal receptors for both the sense of position in the limb and kinaesthesia are the primary afferent fibres that innervate muscle spindles, mechanoreceptors in the joint capsule and cutaneous tactile receptors. Afferent fibres are large and myelinated with a diameter of 12 μm–20 μm. The primary afferent fibres of the muscle spindle are designated as type Ia and the conduction velocities are 72 m/s–120 m/s and respond to both muscle length and rate of length change. Fibres of the same size that arise in skin receptors are called type Aα (2).

The secondary afferent fibres that innervate the muscle spindles possess little sensitivity and therefore provide information only on muscle length and limb position sense. Secondary afferent fibres are myelinated and smaller in diameter (6 μm–12 μm). Secondary afferent fibres are designated type II, also called Aβ, with conduction velocities of 36 m/s–72 m/s.

Golgi tendon organs and joint capsule receptors can also contribute to limb position sense and kinaesthesia. Golgi tendon organs consist of receptors in the tendons that mediate the contractile force or effort of a group of muscle fibres. The afferent fibres of the Golgi tendon organs (type Ib, Aα) are myelinated and large in diameter (12 μm–20 μm), with conduction velocities equal to those of the primary afferent muscle spindle. Joint capsules receive innervation principally from small afferent fibres. These include thinly myelinated type III (Aδ) fibres, which have diameters of 1 μm–6 μm and conduction velocities of 4 m/s–36 m/s, and unmyelinated type IV (C) fibres, which have diameters of 0.2 μm–1.5 μm and conduction velocities of 0.4 m/s–2.0 m/s. Most of these afferent fibres are nociceptors that respond to the extremes of joint position. Joint capsules also receive some innervation from mechanoreceptors that respond to the joint angle. The afferent fibres of these receptors consist of myelinated type II (Aβ) fibres with diameters of 6 μm–12 μm and conduction velocities of 36 m/s–72 m/s (2).

After receiving sensory input from the periphery, the central (proximal) axons of the dorsal root ganglia enter the spinal cord through the medial dorsal root entry zone. Most of the central axonal process will leave the dorsal horn grey matter and enter the dorsal funiculus to form the fasciculus gracilis or fasciculus cuneatus. Fasciculus gracilis carries sensory information associated with the DCML pathway from the lower extremities and terminates and synapses at the nucleus gracilis in the caudal medulla. It is medial and relative to the fasciculus cuneatus and travels along the spinal cord (1).

Fasciculus cuneatus carries sensory information associated with the DCML pathway from the upper extremities. It is located at T6 and above. The fasciculus cuneatus terminates and synapses at the nucleus cuneatus, which is in the caudal medulla. Both the nucleus cuneatus and nucleus gracilis represent the second-order neurons of the DCML pathway. The internal arcuate fibres are axons that emerge ventrally from the dorsal column nuclei and ultimately cross the midline. This is where the DCML pathway decussates. Internal arcuate fibres on the contralateral side of the medulla will join to form the medial lemniscus and travel through the brainstem. There is a somatotopic arrangement in which the ventral fibres arise from the nucleus gracilis and the dorsal fibres arise from the nucleus cuneatus (1).

The medial lemniscus terminates and synapses in the ventral posterolateral (VPL) nucleus of the thalamus with preservation of somatotopy. VPL neurons are third-order neurons; their axons will project laterally out of the thalamus and course somatotopically through the posterior limb of the internal capsule and then terminate in the primary somatosensory cortex of the postcentral gyrus. The tracts of the DCML pathway that begin from the fasciculus gracilis and fasciculus cuneatus to the primary somatosensory cortex have a preserved somatotopic arrangement in which the cervical axons are medial and the sacral axons are lateral (1).

### Sensory Examination

**Figure 1:**
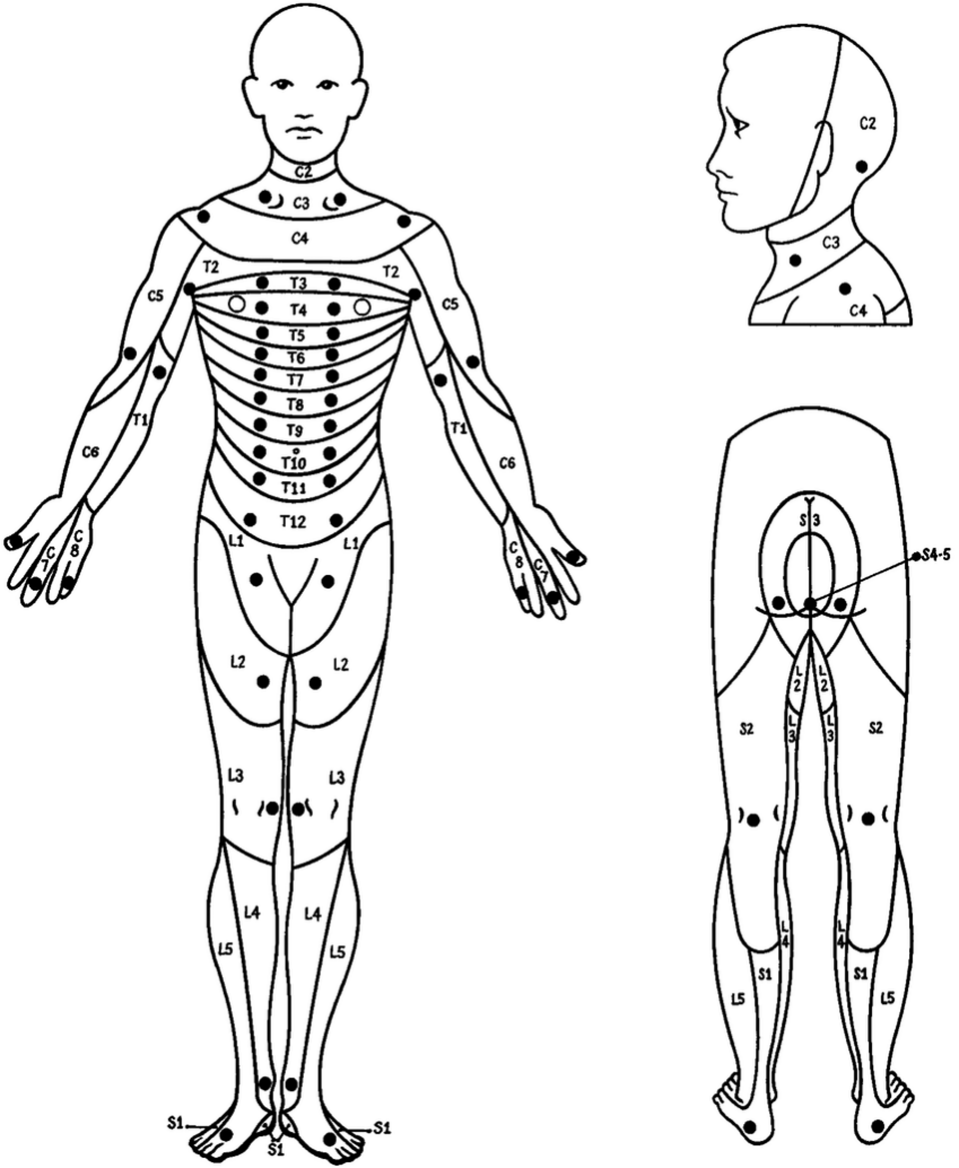
Location of the key sensory points for each dermatome

The dorsal column pathway conveys fine touch, vibration and proprioceptive information. Each dorsal root ganglion and associated spinal nerve arise from a series of embryonic tissue masses called somites. The territory innervated by each spinal nerve is called a dermatome (Figure 1). Dermatomal maps vary between individuals. It also overlaps substantially so that injury to an individual dorsal root does not cause a complete loss of sensation in the relevant skin region; the overlap is more extensive for touch, pressure, and vibration than for pain and temperature. Therefore, the sensation of pain testing provides a more precise assessment of segmental nerve injury than the responses to touch, pressure, or vibration. The segmental distribution of proprioceptors does not follow the dermatomal map but is more closely associated with the muscle innervation pattern.

There are two types of sensation, exteroceptive sensation and proprioceptive sensation. A third sensory modality requires cortical analysis to provide a more complex interpretation of primary sensory information (3).

Exteroceptive sensation (also called superficial sensation): skin and mucous membrane receptors.

Tactile or touch sensation (thermaesthesia):

Anaesthesia: absence of touch appreciation
Hypoesthesia: decrease in touch appreciation
Hyperesthesia: exaggeration of touch sensation, which is often unpleasant

Pain sensation (algesia):

Analgesia: absence of pain appreciation
Hypoalgesia: decrease in pain appreciation
Hyperalgesia: exaggeration of pain appreciation

Temperature sensation, both hot and cold (thermaesthesia):

Thermanalgesia: absence of temperature appreciation
Thermhypersthesia: decrease in temperature appreciation
Thermhyperethesia: exaggeration of temperature appreciation, which is often unpleasant

Proprioceptive sensation (also called deep sensation): receptors that are located in the muscles, tendons, ligaments and joints

Joint position sense (arthraesthesia): absence is described as arthraesthesia
Vibratory sense (pallaesthesia): absence is described as pallaesthesia
Kinaesthesia: perception of muscular motion; usually not measured in a routine clinical evaluation

Cortical sensory functions are interpretative sensory functions that require analysis of individual sensory modalities by the parietal lobes to provide discrimination. Individual sensory modalities must remain intact to measure cortical sensation.

Stereognosis: the ability to recognise and identify objects by feeling them. The absence of this ability is called astereognosis.
Graphesthesia: the ability to recognise symbols written on the skin. The absence of this ability is called graphesthesia.
Two-point discrimination: the ability to recognise simultaneous stimulation by two blunt points. It is measured by the distance between the points required for recognition. Absence is described as such.
Touch localisation (topognosis): ability to localise stimuli to body parts. Topagnosia is the absence of this ability.
Double simultaneous stimulation: the ability to perceive a sensory stimulus when the corresponding areas on the opposite side of the body are stimulated simultaneously. The loss of this ability is called sensory extinction.

### Examination Techniques

Sensory examination is subjective to neurological examination. It must be performed carefully with optimal patient cooperation to achieve reliable results. The patient should be examined in a comfortable and relaxed environment. The examiner should explain each procedure and what is expected from the patient. Before examination, patients should be asked whether they experienced abnormal sensations or numbness or pain in any part of their body. “*Adakah anda mengalami kebas (seghebê), kurang rasa, atau sakit di mana-mana anggota badan?*” If the patient responds positively to these questions, the examination can focus more on the areas involved.

#### Examination of Soft Touch by Semmes-Weinstein Monofilament

The Semmes-Weinstein monofilament test is often used to assess light touch sensation and to determine the minimum stimulation that a subject can feel. Light touch is perceived through receptors on the surface layer of the skin, while firmer pressure is perceived by receptors in the subcutaneous and deep layers of the skin. This test is also known as light touch or deep pressure testing.

**Figure 2:**
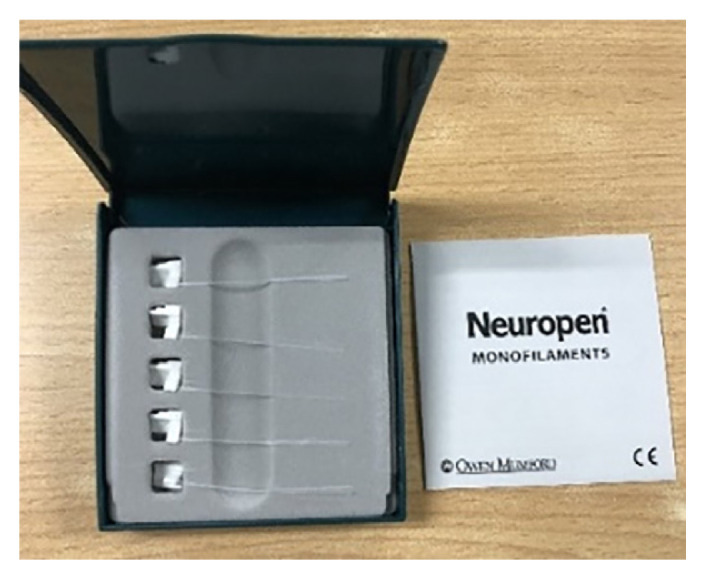
Semmes-Weinstein monofilaments

Semmes-Weinstein monofilaments are rods with a filament mounted at a 90° angle (Figure 2). They are calibrated, single fibre nylon threads with a value ranging from 1.65 to 6.65 that can generate reproducible buckling stress. The higher the value of the monofilament, the stiffer and the more difficult it is to bend. Three monofilaments commonly used to diagnose peripheral neuropathy are 4.17, 5.07 and 6.10. The 5.07/10-g monofilament has been described as the best indicator to determine the loss of protective sensation (4).

Examination of light touch begins with a demonstration of the test on the forehead for normal sensation. The monofilament is then gently stroked along the skin of the midclavicular region of the dermatome C2-S5 bilaterally (5). The patient is asked to indicate if the sensation is equal, increased, decreased or absent compared to the forehead.

Semmes-Weinstein monofilaments also use the pressure perception method as a screening device to identify diabetic patients at risk of foot ulceration (6). A series of monofilaments that range in size (2.83–6.65) are typically used to examine the foot. Monofilaments are applied perpendicular to the skin and are not allowed to bounce, skate or skid across the surface. Patients should close their eyes while being tested. The sensation of pressure using the monofilament should first be demonstrated to the patient on a proximal site, e.g. the upper arm.

The sites of the foot may be examined over the plantar surface of the hallux, third, fifth toe and over the first, third and fifth metatarsal head, medial and lateral sides of the arch and heel, and plantar surface of the distal hallux (big toe). Push the monofilament until it bends, then hold for 1 s–2 s and remove contact from the skin. Thereafter, ask the patient to respond ‘yes’ or ‘no’. The patient should recognise the perception of pressure and identify the correct site; if the patient does not respond, the test at that site should be repeated twice. If there is still no response after repetition, it should be recorded as a negative response. The test will be repeated with a thicker monofilament until the patient can feel the sensation. Areas of callus or thickened skin should always be avoided when testing pressure perception.

Patients without neuropathy should be able to sense the 3.61 monofilament (equivalent to 0.4 g of linear force). The inability to sense monofilaments of ≥ 4.17 (equivalent to 1 g of linear pressure) is considered consistent with neuropathy (large fibre modality). The inability to sense a monofilament of 5.07 (equivalent to 10 g of linear force) is consistent with severe neuropathy and loss of protective sensation (Table 1) (7).

### Examination of Joint Position Sense

The disturbances of proprioception involve the distal joints before the proximal joints; therefore, the most distal joints of each extremity should be tested first. If the testing results of the distal joint are abnormal, more proximal joints should be tested until a normal joint is reached; however, if the distal joint appears normal more tests are rarely required on proximal joints. The third and fourth digits of both the upper and lower extremities are more likely to show early proprioceptive dysfunction, as they are more sparsely innervated than the first, second or fifth digits (3).

Examination of joint position sense is performed by grasping the lateral surfaces of the digit proximal to the joint with the thumb and forefinger, and placing the thumb and forefinger of the other hand distal to the joint and parallel to the plane of movement to avoid the production of pressure stimuli on the surface of the digit. Demonstrate the position of dorsiflexion and ventral flexion (up and down) to the patient and instruct them to respond ‘up’ or ‘down.’ After making sure that the instructions are understood, move the digit through random small up or down movements and ask the patient to respond with eyes closed (3). A total of 10 repetitions are performed and the number of correct trials is recorded (8). Healthy young individuals can detect big toe movements of approximately 1 mm or 2°–3° and in the finger virtually invisible movement, ≤ 1° (9). Sensitivity is less when the joint is in mid-position.

### Examination of Vibratory Sense

Vibratory sense examination is performed using a standardised tuning fork (128 Hz) to produce the vibration stimulus (8). Vibratory sense should first be tested in more distal joints compared to the proximal. If vibration is not perceived on the distal joints, the test should be continued proximally over the bony prominences until it is perceived to determine the extent of the deficit. The tuning fork is struck and placed over the bony prominence and held there until the patient no longer feels the vibration. The patient may describe a feeling of vibration, buzzing, or even gesture with their hands. The tuning fork is placed 90° to the surface of the bony prominence. The tines of the vibrating tuning fork can be placed parallel or perpendicular to the bones, as opposed to the vibrating tuning fork (512 Hz) for the Rinne hearing test. The sound intensity recorded in the tympanic membrane with 512 Hz tuning fork tines is louder in the parallel position by 2.5 dB compared to the perpendicular position for the Rinne test (10).

The sensation may be tested on the big toes, metatarsal heads, malleoli, tibia, anterior superior iliac spine, sacrum, spinous processes of the vertebrae, sternum, clavicle, styloid processes of radius and ulnar, and finger joints (9).

Examination of vibratory sense can be qualitative or quantitative. In one method of qualitative testing, complete loss of vibration sensation is categorised as abnormal. Other qualitative testing methods include comparing the patient’s perception with that of the examiner. This is performed by placing the examiner’s finger on the opposite surface of the joint tested to the tuning fork and noting whether the vibration persists after the patient no longer senses it or by assessing the patient’s threshold against the examiner by applying the tuning fork to their finger (11).

**Figure 3:**
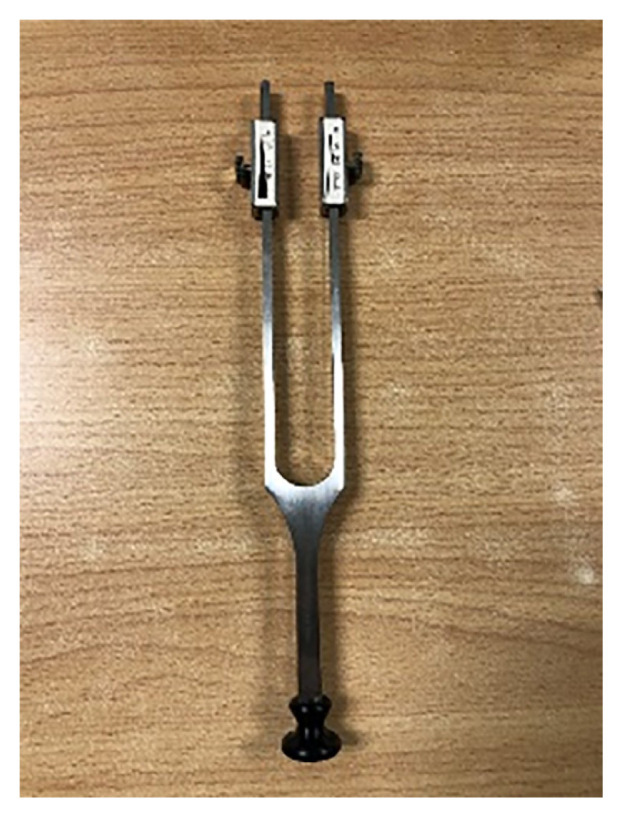
Rydel-Seiffer tuning fork

**Figure 4:**
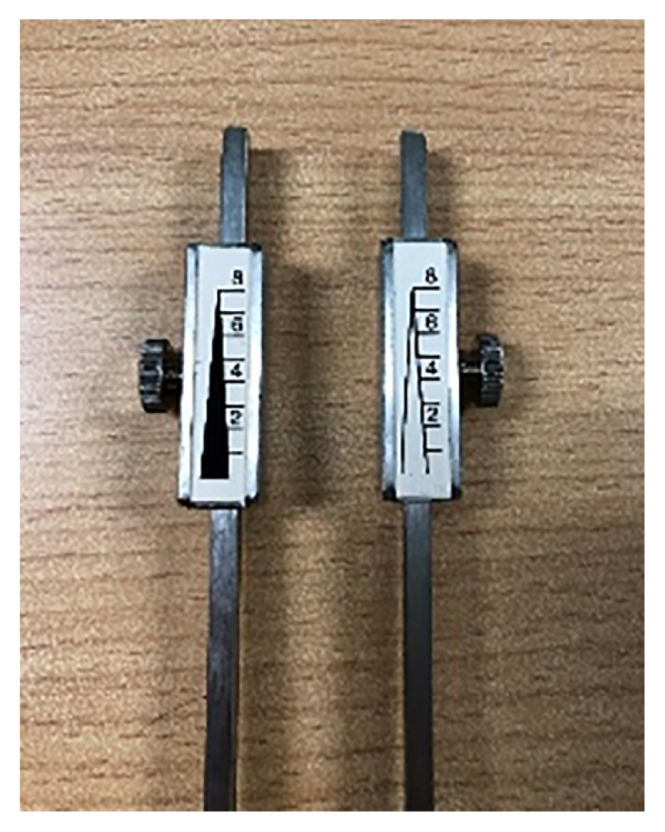
Two arrows and two sets of numbers from 2 to 8

One of the tools to examine the vibration sense is the Rydel-Seiffer tuning fork. The Rydel-Seiffer 64/128 Hz tuning fork is a graduated fork that determines the ability of subjects to discriminate various vibration intensities (Figure 3). The end weights convert the tuning fork from 128 Hz to 64 Hz. The 64 Hz Rydel-Seiffer tuning fork provides a more quantitative assessment of vibratory sensation with a black and white display of two arrows and two sets of numbers from 2 to 8 (Figure 4) (12). Before using the tuning fork, ensure that both dampers are fastened to the tuning fork such that the markings on the back side of the tuning fork line up with the lower edge of the damper, as it is possible for the tuning of the dampers to become loose and move. After the fork is snapped into motion, the prongs start to oscillate, and the illusion of two triangles becomes visible on each damper; as the vibration intensity diminishes, the two triangles move closer together again, and their points of intersection move slowly upwards. The intensity at which the patient no longer detects the vibration is read as the number adjacent to the intersection of the triangles (12).

The normal vibration threshold values (5% lower limit) in healthy controls for the upper and lower extremities are ≥ 6.5 for ≤ 40 years old, ≥ 6.0 for 41 years old-85 years old and ≥ 5.5 for > 86 years old and ≥ 4.5 for ≤ 40 years old, ≥ 4.0 for 41 years old-60 years old, ≥ 3.5 for 61 years old-85 years and ≥ 3.0 for >85 years old, respectively (13).

### Weber Two-Point Discrimination (Static) Test

The two-point discrimination test assesses whether the patient can identify two close points in a small skin area simultaneously and how fine the ability to discriminate is. It is a measure of tactile agnosia, or the inability to recognise these two points despite intact cutaneous sensation and proprioception (14). The aesthesiometer or the circular two-point discriminator are the devices to test.

One or two points are touched in a random sequence along a longitudinal axis in the centre of the fingertip. Examiners must avoid pressing on the calliper with their fingers, thereby artificially increasing the pressure. The American Society for Surgery of the Hand recommends seven correct responses out of 10 for two-point discrimination (15). The smallest distance between the stimuli that is still perceived as two distinct points is measured. The normal distances at which two points can be discriminated at the tip of the tongue, fingertip, dorsum of the fingers, palm and dorsum of the hand are 1 mm, 2 (4 mm), 4 (6 mm), 8 (12 mm) and 20 (30 mm), respectively (9).

For moving two-point discrimination, the aesthesiometer was moved in a proximal to distal direction. The tip of the aesthesiometer is 10 mm apart and then passed distally along with the finger pulp. The patient must distinguish between one or two tips. If the patient responds correctly to seven out of ten passes, then the gap between the tips is narrowed (14). The moving two-point discrimination test is also known as the Dellon’s two-point discrimination test.

### Baseline Aesthesiometer, Two-Point Discriminator (Figure 5)

This is to evaluate cutaneous sensitivity and touch threshold. This aesthesiometer measures up to 10 cm of discrimination. The two tips of the instrument are applied to the skin simultaneously with the tips spread apart. The two tips are gradually brought closer together until the patient makes errors and perceives the stimuli as one. The intersection of the arm with the line on the other arm shows the distance between the two points (10 cm, maximum distance and 3 mm, minimal distance).

### Rhomberg Test

The Romberg test is also a test of joint position sense. The patient is asked to remove their shoes and stand with both feet together. The patient is asked to hold his/her arms close to the body or cross them in front of the body. The first stage of the test involves asking the patient to keep their eyes open while the examiner assesses the patient’s body movement relative to balance (16).

The second stage involves instructing the patient to stand erect with their eyes closed while the examiner notes any balance impairment for 1 min. Swaying of the body may be observed. However, this indicates that the proprioceptive correction of balance is due to a lack of visual or vestibular compensation. The Rhomberg test is positive when the patient loses balance with their eyes closed. Loss of balance can be defined as increased body sway, foot movement in the direction of fall or fall (16).

## Conclusion

Examination of the dorsal column is important for the diagnosis of peripheral and central nervous system diseases. It is important to have a proper technique of examination to reduce the discrepancy between each examiner. We hope that students can adhere to these techniques and apply them in their daily neurological assessment.

The link to the examination video is available at the following:

Soft touch examination: https://youtu.be/dcPzgx5kzjY
Joint position sense examination: https://youtu.be/zmOdSlkMFA0
Vibratory sense examination: https://youtu.be/b-4cpN8gyNQ
Two-point discrimination test: https://youtu.be/5EhlG9l2wHo
Rhomberg test: https://youtu.be/FmRB3z39M6g

## Figures and Tables

**Figure 5:**
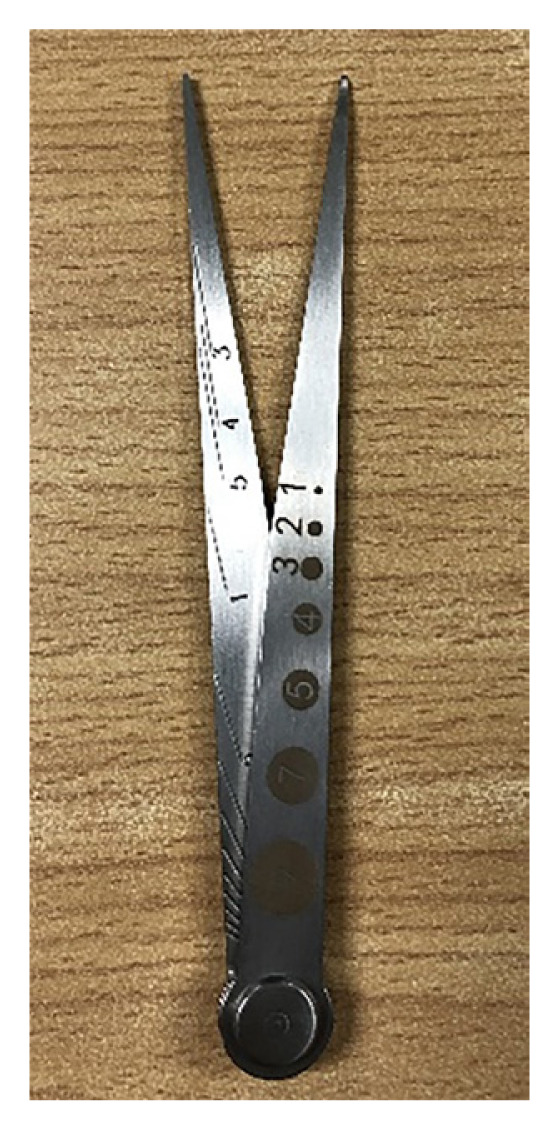
Baseline aesthesiometer

**Table 1:** Monofilaments size, its equivalent force in gram and its significant

Evaluator size	Target force (g)	Plantar threshold
1.65	0.008	Normal
2.36	0.020	
2.44	0.040	
2.83	0.070	
3.22	0.160	
3.61	0.40	
3.84	0.6	Diminished light touch
4.08	1.0	
4.17	1.4	
4.31	2.0	
4.56	4	Diminished protective sensation
4.74	6	
4.93	8	
5.07	10	Loss of protective sensation
5.18	15	
5.46	26	
5.88	60	
6.10	100	
6.45	180	
6.65	300	Deep pressure sensation only

## References

[1] Al-Chalabi M, Reddy V, Alsalman IAM (2018). Neuroanatomy, posterior column (dorsal column).

[2] Gilman S (2002). Joint position sense and vibration sense: anatomical organisation and assessment. J Neurol Neurosurg Psych.

[3] Bigley GK, Walker HK, Hall WD, Hurst JW (1990). Sensation. Clinical methods: the history, physical, and laboratory examinations.

[4] Dros J, Wewerinke A, Bindels PJ, van Weert HC (2009). Accuracy of monofilament testing to diagnose peripheral neuropathy: a systematic review. Ann Fam Med.

[5] Kocarev M, Watkins E, McLure H, Columb M, Lyons G (2010). Sensory testing of spinal anaesthesia for caesarean section: differential block and variability. Int J Obstet Anesth.

[6] Kumar S, Fernando DJ, Veves A, Knowles EA, Young MJ, Boulton AJ (1991). Semmes-Weinstein monofilaments: a simple, effective and inexpensive screening device for identifying diabetic patients at risk of foot ulceration. Diabetes Res Clin Prac.

[7] Tanenberg RJ, Donofrio PD, Bowker JH, Pfeifer MA (2008). Chapter 3: neuropathic problems of the lower limbs in diabetic patients. Lein and O’Neil’s the diabetic foot.

[8] Prabhakar AT, Suresh T, Kurian DS, Mathew V, Ahmed Shaik AI, Aaron S (2019). Timed vibration sense and joint position sense testing in the diagnosis of distal sensory polyneuropathy. J Neurosci Rural Pract.

[9] Campbell WW, Barohn RJ (2020). DeJong’s the neurologic examination.

[10] Butskiy O, Ng D, Hodgson M, Nunez DA (2016). Rinne test: does the tuning fork position affect the sound amplitude at the ear?. J Otolaryngol Head Neck Surg.

[11] Lai S, Ahmed U, Bollineni A, Lewis R, Ramchandren S (2014). Diagnostic accuracy of qualitative versus quantitative tuning forks: outcome measure for neuropathy. J Clin Neuromuscul Dis.

[12] Panosyan FB, Mountain JM, Reilly MM, Shy ME, Herrmann DN (2016). Rydel-Seiffer fork revisited: beyond a simple case of black and white. Neurology.

[13] Martina IS, van Koningsveld R, Schmitz PI, van der Meche FG, van Doorn PA (1998). Measuring vibration threshold with a graduated tuning fork in normal aging and in patients with polyneuropathy. J Neurol Neurosurg Psychiatry.

[14] Rea PM, Rea PM (2015). Chapter 8. Spinal tracts—ascending/sensory pathways. Essential clinical anatomy of the nervous system.

[15] Dumontier C, Tubiana R, Weinzweig J (2010). Chapter 115—Physical examination of the hand. Plastic surgery secrets plus.

[16] Forbes J, Munakomi S, Cronovich H (2022). Romberg test.

